# Change in glycaemic control with structured diabetes self-management education in urban low-resource settings: multicentre randomised trial of effectiveness

**DOI:** 10.1186/s12913-023-09188-y

**Published:** 2023-02-24

**Authors:** Roberta Lamptey, Mary Amoakoh-Coleman, Mary Moffett Barker, Samuel Iddi, Michelle Hadjiconstantinou, Melanie Davies, Daniel Darko, Irene Agyepong, Franklyn Acheampong, Mary Commey, Alfred Yawson, Diederick E. Grobbee, George Obeng Adjei, Kerstin Klipstein-Grobusch

**Affiliations:** 1 Polyclinic/ Family Medicine Department, Korle Bu Teaching Hospital, Accra, Ghana; 2 Department of Community Health, University of Ghana Medical School, Accra, Ghana; 3 Julius Center for Health Sciences and Primary Care, Julius Global Health, University Medical Center Utrecht, Utrecht University, Utrecht, The Netherlands; 4 Department of Epidemiology, Noguchi Memorial Institute for Medical Research, University of Ghana, Accra, Ghana; 5 Diabetes Research Centre, University of Leicester, Leicester, UK; 6 NIHR Leicester Biomedical Research Centre, Leicester, UK; 7 Department of Statistics and Actuarial Science, University of Ghana, Legon, Ghana; 8 Diabetes Research Centre, University of Leicester, Leicester, UK; 9 NIHR Leicester Biomedical Research Centre, Leicester, UK; 10 Family Medicine Department, Nyaho Medical Center, Accra, Ghana; 11 Department of Physician Assistantship Studies, Central University, Prampram, Ghana; 12 Faculty of Public Health, Ghana College of Physicians and Surgeons, Accra, Ghana; 13 Office of Research, Korle Bu Teaching Hospital, Accra, Ghana; 14 Non-Communicable Diseases Control Programme, Ghana Health Service, Accra, Ghana; 15 Divison of Epidemiology and Biostatistics, School of Public Health, Faculty of Health Sciences, University of Ghana, Accra, Ghana; 16 Centre for Tropical Clinical Pharmacology and Therapeutics, University of Ghana Medical School, Accra, Ghana; 17 Office of Research Innovation and Development, University of Ghana, Legon, Ghana; 18 Division of Epidemiology and Biostatistics, School of Public Health, Faculty of Health Sciences, University of the Witwatersrand, Johannesburg, South Africa; 19 Korle Bu Teaching Hospital, Guggisberg avenue, Accra, Ghana

**Keywords:** Diabetes, DSME, HbA1c, Self-care, Low-resource

## Abstract

**Background:**

In high-resource settings, structured diabetes self-management education is associated with improved outcomes but the evidence from low-resource settings is limited and inconclusive.

**Aim:**

To compare, structured diabetes self-management education to usual care, in adults with type 2 diabetes, in low-resource settings.

Research design and methods.

**Design:**

Single-blind randomised parallel comparator controlled multi-centre trial.

Adults (> 18 years) with type 2 diabetes from two hospitals in urban Ghana were randomised 1:1 to usual care only, or usual care plus a structured diabetes self-management education program. Randomisation codes were computer-generated, and allotment concealed in opaque numbered envelopes. The intervention effect was assessed with linear mixed models.

Main outcome: Change in HbA1c after 3-month follow-up.

Primary analysis involved all participants.

Clinicaltrial.gov identifier:NCT04780425, retrospectively registered on 03/03/2021.

**Results:**

Recruitment: 22^nd^ until 29^th^ January 2021.

We randomised 206 participants (69% female, median age 58 years [IQR: 49–64], baseline HbA1c median 64 mmol/mol [IQR: 45–88 mmol/mol],7.9%[IQR: 6.4–10.2]). Primary outcome data was available for 79 and 80 participants in the intervention and control groups, respectively. Reasons for loss to follow-up were death (n = 1), stroke(n = 1) and unreachable or unavailable (n = 47). A reduction in HbA1c was found in both groups; -9 mmol/mol [95% CI: -13 to -5 mmol/mol], -0·9% [95% CI: -1·2% to -0·51%] in the intervention group and -3 mmol/mol [95% CI -6 to 1 mmol/mol], -0·3% [95% CI: -0·6% to 0.0%] in the control group. The intervention effect was 1 mmol/mol [95%CI:-5 TO 8 p = 0.726]; 0.1% [95% CI: -0.5, 0.7], p = 0·724], adjusted for site, age, and duration of diabetes.

No significant harms were observed.

**Conclusion:**

In low-resource settings, diabetes self-management education might not be associated with glycaemic control. Clinician’s expectations from diabetes self-management education must therefore be guarded.

**Supplementary Information:**

The online version contains supplementary material available at 10.1186/s12913-023-09188-y.

## What is already known on this topic?

- In high-resource settings, structured diabetes self-management education is associated with improved outcomes.

## What this study adds?

- There was no between group difference in mean HbA1c at 3 months following a 6-hour structured DSME intervention.
- HbA1c decreased by 9 mmol/mol [95%CI:-13 to-5, *p*<0·001]; 0·9% [95% CI: - 1.2 to -0·5,*p*<0·001] in the intervention arm.
- HbA1c decreased by-3 mmol/mol [95%CI:-6 to 1, *p*=0·172]; 0·3% [95% CI: -0·6 to 0·0, *p*=0·002] in the control arm.

## What are the implications of the study?

In low-resource settings, the effect size of structured diabetes self-management education on glycaemic control may be limited and thus, clinician’s expectations from diabetes education must be guarded.

## Background

Diabetes is a long-standing epidemic with over half a billion adults affected globally. [1] In Ghana, the overall prevalence of diabetes among the general adult population is 7% [2]. Diabetes is a leading cause of mortality in Accra, the capital of Ghana. [3] However, people living with diabetes (PLD) often have limited knowledge about self-management. [4] This could contribute to poor glycaemic control.

Self-care is essential for PLD. This underpins the need for self-management education. In high-income countries, structured diabetes self-management education (DSME) programmes such as, the Diabetes Education and Self-Management for Ongoing and Newly Diagnosed (DESMOND) program, are associated with improved outcomes [5, 6]. In low- and middle-income countries, the association between structured diabetes education and diabetes outcomes is however inconclusive [7, 8].

Indeed, DSME services are limited in Ghana, a low-middle-income country. ^9^ Due to the high disease burden, determinants of glycaemic control are prioritised in Ghana’s national health research agenda [9]. We therefore sought to investigate the effect of structured DSME on glycaemic control in two low-resource settings in Accra, Ghana[10].

## Methods

### Study design and approval

A multicentre, parallel-group, single-blind randomised controlled trial was conducted at two hospitals (WGMH and KBTH) in Accra, Ghana. Adults living with type 2 diabetes were randomised 1:1 to structured DSME plus usual care, or usual care only.

### Ethical approval

Ethical approval was provided by the Ghana Health Service Ethics Review Committee (protocol ID no: GHS-ERC 009/11/20), and the Institutional Review Board of KBTH (protocol ID no: KBTH-IRB 000,175/2021).

### Study participants and study setting

Eligibility criteria included aged 18 years or above, ability to participate in activities in a group setting, known to have T2DM, and not known to have chronic kidney disease or sickle cell disease.

The study was conducted between January-May 2021, at two public primary facilities in Accra, Ghana. Potential participants were identified by searching electronic medical records of the study sites. Using attendance records, trained staff called all potential participants meeting eligibility criteria and invited them to participate. Participants, who expressed interest in the study, were given appointments for a screening visit at the study sites. Participants were recruited from 22^nd^ to 29^th^ January 2021.

Prior to any study procedures, all participants gave written informed consent in person. Participants received reimbursement for travel costs and time.

### Randomisation and masking

Participants were randomly assigned either to usual care, or usual care plus intervention. Usual care at KBTH polyclinic consisted of informal brief education given by doctors whilst consulting. At WGMH, usual care consisted of unstructured group education, lasting approximately 30 min, delivered on clinic days; by nurses.

Enrolled patients were randomised the same day. Stratified randomisation, by participant age (< 40 years or ≥ 40 years), was carried out in variable blocks with the aid of a centralised computer-generated sequence. Each patient randomised had an electronically generated unique identification number matching the assigned study arm. Allotment was concealed in sequentially numbered opaque envelopes and sealed. Care providers at both hospitals were blinded.

### Procedures

#### Intervention

The intervention tested was a structured DSME program which had been adapted from DESMOND: EXTENDing availability of self-management structured education programmes for people with type 2 Diabetes in low-to-middle income countries (EXTEND). EXTEND has been piloted in Malawi and Mozambique [11]. DESMOND is a cost-effective structured DSME program, originally developed in the United Kingdom [6, 12, 13].

We further culturally adapted EXTEND to the Ghanaian community; citing local cuisine and contextualising examples [14].

Five community health nurses and one medical officer were trained virtually, by DESMOND trainers to deliver the intervention. The intervention was delivered in-person, while observing all coronavirus infectious disease-2019 (COVID-19) protocols. The intervention consisted of one session of structured DSME, delivered by two educators to groups of six to ten participants in one day, over 6 h. The delivery of the intervention was completed within 2 weeks of randomisation. The intervention was delivered by providers not directly involved in patient care.

### Follow-up intervals and assessments

**Fig. 1:**
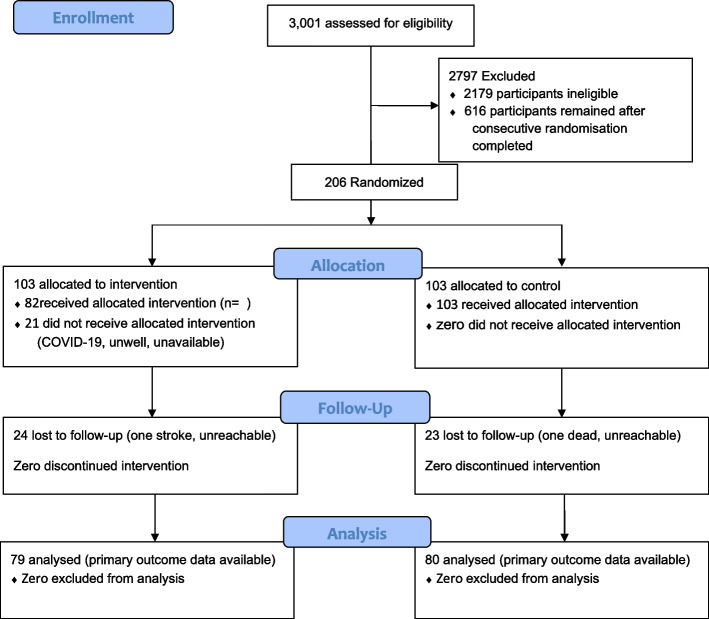
Trial profile

The first 206 patients were consecutively randomised 1:1 either to structured DSME plus usual care or usual care only (Fig. 1). At randomisation (baseline) and three months after randomisation, participants completed an interviewer-administered questionnaire and underwent a clinical assessment. Baseline data was collected on 26^th^ and 27^th^ January 2021 at KBTH, and on 22^nd^ and 29^th^ January 2021 at WGMH. Follow-up data was gathered on 14^th^ and 15^th^ May 2021 at KBTH, and on 20^th^ and 22^nd^ May 2021 at WGMH. The final on-site data collection was conducted at WGMH, on 12^th^ June 2021.

Despite prior acceptance of the invitation to participate in endline data collection, some participants failed to show up by the trial end date. Specifically, 71 participants in the intervention group and 78 in the control group completed follow-up at 3 months. Ten participants completed follow-up between 3 to 5 months: eight in the intervention and two in the control group.

### Outcomes

The primary outcome was change in HbA1c after 3-month follow-up. HbA1c was assessed centrally in an accredited laboratory, adhering to international criteria, set out according to International Organisation for Standardisation standards (ISO Standard 15,189:2012). HbA1c measurement was conducted using the turbidimetric inhibition immunoassay method, with a ROCHE COBAS intergra 400 plus analyser.

Secondary outcomes were, changes in clinical, psychological, and self-care variables. Specifically, the clinical outcomes were change in weight, waist circumference, and blood pressure respectively; the psychological outcomes were changes in diabetes-related distress scores[15] and WHO quality of life scores respectively[16]; and the self-care outcome was change in diabetes self-care activities (SDSCA) scores [17].

The SDSCA scale assesses the level of self-care in five domains, namely diet, exercise, glucose monitoring, foot care, and smoking. The WHO Qol Bref instrument assess the quality of life. We assessed diabetes-related distress with, the problem areas in diabetes-5 (PAID-5) scale. Increasing scores indicate increasing distress; scores of 8 or more suggest diabetes-related emotional distress [15].

At follow-up, we assessed adverse events using a standardised questionnaire.

### Statistical analysis

#### Sample size calculation

Mean reductions in HbA1c, between baseline and follow-up, were assumed to be 0 mmol/mol (0·0%) in the usual care group, and 4·8 mmol/mol (0·5%) in the intervention group [8]. We assumed mean baseline HbA1c at KBTH to be 72 mmol/l sd 8 (8.7% sd 2.7). To achieve 80% power (p < 0·05, two-tailed) to detect a (0·5%) difference in mean HbA1c between the intervention and control group, a sample size of 148 participants (74 per group) was required. We assumed 20% loss to follow-up, thus 89 participants per arm were required after screening. It was assumed that 20% of recruited participants would be ineligible at screening, therefore 213 participants (107 per arm) needed to be recruited.

At the time of recruitment, the COVID-19 epidemic was unfolding with vaccines not yet available. Considering the uncertainties surrounding the epidemic, we decided to assess all potentially eligible participants. Subsequently we consecutively randomised the first 206 of those meeting the eligibility criteria.

### Comparative analysis

Baseline sociodemographic, clinical, psychosocial, and self-care variables were summarised using median (interquartile range [IQR]) for continuous variables and counts (percentages) for categorical variables. All analysis was by intention to treat.

The study was powered to detect the average response to the intervention, between the two groups. We therefore fitted a model to determine the difference in HbA1c at the end of the study. Specifically, we used linear mixed models to assess the intervention effect for the primary outcome. The models were adjusted for key prognostic factors, site, treatment arm, and follow-up duration. The model was parameterised as follows:

$${Y}_{ij}={\beta }_{0}+\sum_{k=1}^{p-1}{\beta }_{k}{X}_{kij}+{{\beta }_{p}T}_{ij}+{b}_{0i}+{\epsilon }_{ij}$$

where ${Y}_{ij}$ represents the outcome for subject $i$ measured at ooccasion $j=\mathrm{1,2}$, ${X}_{kij}$ is the kth explanatory variable including an indicator for the intervention, ${T}_{ij}$ is the time variable, ${\beta }_{k+1}$ are the fixed-effects,

${b}_{0i}\sim N\left(0,{\sigma }_{b}^{2}\right)$ is the random-effect accounting for within-subject correlation and ${\epsilon }_{ij}\sim N(0, {\sigma }^{2})$ is the pure error term.

All analysis was conducted in R statistical package and statistical significance was set at two-sided *p* < 0·05.

The trial is registered on clinical trials.gov; registration number NCT04780425.

## Results

All 206 participants randomised were included in the analysis (Table 1). Importantly, over 10% (*N* = 21) of participants randomised to the intervention did not receive the intervention. At the time of delivery of the intervention, three participants were unwell including one who had tested positive for COVID-19. One other participant declined the invitation to the structured DSME session, for fear of contracting COVID-19. Aside these four, seven participants were unavailable on the day of the intervention. Four had travelled out of Accra, two were engaged and one was babysitting. The remaining ten out of these 21 participants were unreachable by telephone.

**Table 1:** Descriptive characteristics of participants, by intervention group, median(Q1^*^,Q3^†^) for continuous or N^‡^ (%)^#^ for categorical variables

	**Control (*N* = 103)**	**Intervention (*N* = 103)**	**Total (*N* = 206)**
Hospital Site			
N^‡^	103	103	206
Korle bu Teaching Hospital	55 (53%)	55 (53%)	110 (53%)
Weija Gbawe Municipal Hospital	48 (47%)	48 (47%)	96 (47%)
Sex			
N-Miss^¶^	0	1	1
N	103	102	205
Male	32 (31%)	32 (31%)	64 (31%)
Female	71 (69%)	70 (69%)	141 (69%)
Age(years)			
N-Miss	0	1	1
N	103	102	205
Median (Q1^¶^, Q3^**#**^)	57 (50, 64)	59 (49, 64)	58 (49, 64)
Education			
N-Miss	1	0	1
N	102	103	205
None	11 (11%)	10 (10%)	21 (10%)
Primary	17 (16.7%)	16 (16%)	33 (16%)
Middle or higher	74(73%)	77 (75%)	151 (74%)
Occupation			
N	103	103	206
Professionals with degrees	5 (5%)	8 (8%)	13 (6%)
other occupation	45 (44%)	60 (58%)	114 (55%)
educated youth, unemployed	44 (43%)	35 (34%)	79 (38%)
Total Income(Dollars)			
N-Miss	10	8	18
N	93	95	188
Median (Q1, Q3)	40(20, 90)	50 (23, 100)	50 (20, 100)
Duration of diabetes (years)			
N	103	103	206
Median (Q1, Q3)	5 (3, 10)	5 (2, 10)	5 (3, 10)
HbA1c** (%)			
N	103	103	206
Median (Q1, Q3)	7.6 (6.3, 6.6)	8.2 (6.5, 10.6)	8 (6, 10)
HbA1c (mmol/mol)			
N	103	103	206
Median (Q1, Q3)	58 (45, 82)	67 (47, 92)	63 (45, 88)

^*^Q1 is the lower quartile. ^†^ Q3 is the upper quartile. ^‡^N is number of observations

^#^% is percentage of observation. ^¶^ N-Miss is number of missing observations ^**^HbA1c is glycated Haemoglobin

Altogether, 22% (*N* = 46) of the participants randomised were lost to follow-up. The commonest reason for inability to provide follow-up data for the primary outcome was “unreachable by telephone” or “unavailable” during the period allocated for blood sampling. Only 1% (*N* = 2) of the participants randomised had alternative reasons. These reasons are depicted in the trial profile (Fig. 1).

### Baseline Characteristics

The baseline characteristics show high WHO Qol scores, despite low incomes, low literacy, and high unemployment levels (Table 1 /Supplementary Table 1). Furthermore, in most of the domains of the SDSCA score, the median score was once weekly. At baseline median HbA1c values were slightly higher in the intervention group than in the control group. (Table 1 and Supplementary Table 1). Notwithstanding sub-optimal baseline HbA1c values and low SDSCA scores, the overall median baseline PAID score was below eight.

### Primary outcome

At endline, HbA1c decreased within both groups:-0·9% in the intervention group and -0·3% in the control group. Although this decrease was greater in the intervention group than in the control group, the difference between groups was not significant (Table 2). The primary outcome failed to reach significance. There was insufficient evidence that the intervention had an effect on HbA1c (Supplementary Figs. 1 and 2).

**Table 2:** Effect of the intervention (structured DSME*) on the primary outcome (average change in HbA1c†) after 3-months follow-up.^‡^

Fixed Effects	Unit: mmol/mol			Units: %		
**Parameters**	**Coefficients**	**95% CI**	**P-value**	**Coefficients**	**95% CI**	**P-value**
Intercept	72	56 to 89	< .001	8.8	7.3 to 10.3	< .001
Site (WGMH)^¶^	9	2 to 15	0.007	0.8	0.2 to 1.4	0.006
Diabetes duration (years)	1	0 to 1	0.023	0.1	0.0 to 0.1	0.021
Age(years)	0	-1 to 0	0.081	0.0	-0.1 to 0.0	0.071
Arm (Intervention)	1	-5 to 8	0.726	0.1	-0.5 to 0.7	0.724
Follow-up interval^**#**^	-2	-3 to -1	< .001	-0.2	-0.3 to -0.1	< .001
	**Random Effects**					
**Parameter**	**SD**			**SD**		
Intercept	21			1.9		
Residual	13			1.5		
Intraclass correlation	0.7			0.6		

^*^DSME is diabetes self-management education intervention. The intervention tested was an adapted version of an evidence based structured DSME: Diabetes Self-Management Education for New and ON-going Diabetes (DESMOND) [6, 14]. The comparator was usual care

^†^ HbA1c is glycated Haemoglobin. ^‡^Data are presented as coefficient estimates from linear mixed models

^¶^WGMH is Weija Gbawe Municipal Hospital site. The comparator was Korle Bu Teaching Hospital Polyclinic

^**#**^ Participants were followed for at least three months

### Secondary outcomes

Similarly, there was insufficient evidence that the intervention had an effect on any of the secondary outcomes except for an improvement in physical health. The difference in physical health between the intervention and control was 3 (*p* = 0.035)( Supplementary Table 4d). The within-group differences for both arms were also not significant for most clinical variables and psychological variables. The mean waist circumference of the control group was 1 cm higher relative to baseline (*p* = 0.015) while in the intervention arm, the mean waist circumference remained unchanged (*p* = 0.249). Similarly, the average systolic blood pressure increased by 5 mmHg (*p* = *0.143*) in the control arm while in the intervention arm the mean systolic pressure remained unchanged (*p* = 0.249) ( Supplementary Tables 3c/3d). On the contrary, these differences were significant for self-care activities namely foot care, exercise, and diet. In the control arm, the mean number of days in a week, that participants inspected the inside of their shoes increased by 1 day at endline (*p* = 0.003). Correspondingly, among the intervention, the mean number of days per week increased by 2 (*p* < *0.001*). ( Supplementary Tables 3a / 3b). The interaction term between the follow-up interval and intervention arm did not reach significance for any of the secondary outcomes.

### Adverse events

No significant harms were observed. One participant however, had to be treated for symptomatic hypoglycaemia during delivery of the intervention. The participant’s medications included human insulin.

## Discussion

Our aim was to study the association between structured DSME, and glycaemic control. Our results show that, in people living with diabetes (PLD) in resource-constrained settings, structured DSME may not be associated with change in HbA1c at 3-months. A clinically relevant reduction in HbA1c was observed in the intervention group but not in the control group.

Reduction in HbA1c is associated with less micro-vascular complications and may also reflect better self-management routines. In sub-Saharan Africa, many PLD have poor self-management skills and poor diabetes outcomes [1, 18]. DSME provides knowledge for effective self-management and is thus, especially important for PLD in sub-Saharan Africa. Unfortunately, in resource-constrained settings translating self-management skills into practice can be challenging.

There are multiple and complex barriers to self-management in low-resource settings. These barriers include irregular diabetes supplies, financial constraints, low health literacy and culture [19]. In the African context, culture is a significant barrier to adhering to self-management recommendations [20, 21]. Complex cultural belief systems are particularly challenging for Ghanaians living with diabetes [10, 22].

The DSME intervention we tested was culturally tailored for the Ghanaian population, and linguistically suited to low-literacy levels. This may potentially explain the clinically relevant and significant reduction in HbA1c within the intervention group. HbA1c is dependent on self-management routines. The changes in the summary of diabetes self-care activities scores suggest an improvement in self-management routines within the intervention group.

Our findings align with findings from most previous studies [7, 23, 24]. In a recent systematic review of randomised controlled trials investigating structured DSME in PLD in Africa, seven out of the nine studies found no association between structured DSME and HbA1c. Furthermore, sub-group analysis revealed that, characteristics of structured DSME interventions such as cultural tailoring, duration, and intensity were not associated with HbA1c.

The studies included in this systematic review share some characteristics with our study and this may contribute to the congruence in the findings [7]. In the studies included in the review, the structured DSME interventions tested were culturally tailored, delivered mostly by nurses and the comparator was usual clinical care. The minimum mean baseline HbA1c was over 8%.[7]

The baseline mean HbA1c was over 10% in Hailu et al.’s study among participants in Ethiopia. Additionally, 50% of their study population had lived with diabetes for over 10 years and participants received six sessions of DSME over a 9-month period [24]. Compared to participants in our study, participants in Hailu et al.’s study had relatively higher baseline HbA1c values and longer study duration. Yet, the findings from both studies are congruent. The study by Gathu et al. in Kenya is another study included in the systematic review [7]. It was conducted in a single private facility among participants with low deprivation. Our study was conducted in two public facilities among participants with high deprivation. Again, despite the differences, the findings from the two studies are congruent.

In summary, despite the case-mix variation between studies there is homogeneity in the estimates of the association between structured DSME and glycaemic control in PLD in Africa [7, 23, 24].

Our findings of a null association between structured DSME and glycaemic control are inconsistent with studies from Kuwait, Nigeria, and our recently published systematic review of structured DSME in low- middle-income countries[8, 25, 26]. Our study population shares similar characteristics with the study by Alibrahim et al. undertaken in a primary care setting in Kuwait. Alibrahim et al. observed that, single 1-h small group DSME sessions were associated with 1.7% reduction in HbA1c at 12 months [25]. Consistent with these findings from Kuwait, Essien et al. also reported a 1.8% reduction in HbA1c at six months among participants in Nigeria living with type 1 or type 2 diabetes [26].

Our findings on HbA1c are consistent with those of Davies et al. in a study undertaken in the UK[6], but differ from the findings from Mozambique and Malawi[11], albeit in all three studies, the intervention tested was underpinned by the same psychological theories of learning. Our study, and the study conducted by Davies et al. were randomised control trials; this design maximises internal validity and permits causal inferences. Brady et al.[11], performed a feasibility study (with no control group) on a purposively selected sample of 50 people, thus limiting the ability to assess the relationship between structured DSME and HbA1c. Davies et al. included only participants who were newly diagnosed with diabetes and used a cluster randomised design. This design may limit contamination between the study groups.

In resource constrained-settings intervention contact time can limit sustainability and scalability of a structured education program. It is not clear what the relation between intervention contact time and reduction in HbA1c is. DSME Interventions with contact time greater or equal to 10 h, have been shown to be associated with significant reductions in HbA1c in a systematic review of over 100 varieties of DSME programs [27]. However, in a systematic review focusing on studies in African American populations, no association between HbA1c and intervention contact, and provider time was observed [28]. In our study the total contact time was desirably short although the primary outcome did not reach significance.

For most participants in our study, health literacy was low, and the monthly cost of care was greater than half of their monthly income. The average cost of managing one person with diabetes in a clinic in Accra in 2009 was about US$ 28 monthly [29]. There is a positive linear association between health literacy and socio-economic variables [29]. The low income levels, combined with low literacy, possibly denote high deprivation and this could have biased the association between HbA1c and structured DSME.

Our inability to standardise usual care between groups is also a potential source of bias. Due to the nature of the intervention, it was not possible to blind assessors, and this might be responsible for ascertainment bias. However, the primary outcome, change in HbA1c was an objective outcome thus limiting the risk of bias. Higher baseline HbA1c values in the intervention group, may indicate that those in the intervention group had more advanced disease. This difference could also have biased our estimate. Reassuringly these differences were not statistically significant; pointing to a robust randomization. Stratification on baseline HbA1c would have resulted in more balanced groups. At the time of randomisation however,, baseline HbA1c values were not known.

The randomised controlled trial design, and the use of a culturally adapted intervention are strengths of this study. This design increases the internal validity of the estimate of the effect of structured education on glycaemic control in the population.

The study was set in Ghana, in two public health facilities, where the national health insurance scheme is the main means of healthcare financing. Our findings may therefore not be generalisable to community-based interventions or private facilities. We excluded participants who were not ambulant, could not participate in group activities and who were not primarily responsible for their care, thus further limiting the generalisability of our findings.

## Conclusion

Glycaemic control was not associated with DSME in this study, although the reduction in HbA1c was larger in the intervention group compared to usual care. Ideally, DSME equips individuals with skills for decision making and taking action. ^30^ Deprivation as commonly pertains in resource-constrained settings in SSA limits options and thus, the possibility of taking action.

We thus recommend larger cluster randomised studies, with longer duration of follow-up, which focus on enumerating the effect of structured DSME on glycaemic control, in resource-constrained settings.

## Supplementary Information

**Additional file 1.** Supplementary figure 1

**Additional file 2.** Supplementary figure 2

**Additional file 3.** Supplementary tables

## Data Availability

The datasets used and/or analysed during the current study are available from the corresponding author on reasonable request.

## Abbreviations

DSME: Diabetes Self-management education
PLD: Persons living with diabetes
KBTH: Korle Bu Teaching Hospotal
WGMH: Weija Gbawe Municipal Hospital
GHS: Ghana Health Service
DESMOND: Diabetes Self-Management Education for On-going and Newly Diagnosed
EXTEND: EXTENDing availability of self-management structured education programmes for people with type 2 Diabetes in low-to-middle income countries
COVID-19: Corona virus infectious diseases-19
HbA1c: Glycated Haemoglobin
SDSCA: Summary of Diabetes Self-care Activities Measure
PAID: Problem Areas in Diabetes
WHO QoL: WHO Quality of Life
IRB: Institutional Review Board
ERC: Ethical Review Committee
IQR: Inter-quartile range
Q1: First quarter
Q3: Third quartile
