# Supplementary material for: Change in glycaemic control with structured diabetes self-management education in urban low-resource settings: multicentre randomised trial of effectiveness
Source: BMC Health Serv Res. 2023 Feb 24;23:199. doi: 10.1186/s12913-023-09188-y (PMC9957611; doi:10.1186/s12913-023-09188-y)
Supplement: Supplementary file 1 — Additional file 1. Supplementary figure 1 [file 12913_2023_9188_MOESM1_ESM.docx]

| **Supplementary Table 1 (a).** Baseline characteristics of participants, by intervention group, median(Q1*,Q3†) for continuous or N^‡^ (%)^#^ for categorical variables. | | | |
| --- | --- | --- | --- |
|  | **Control (N=103)** | **Intervention (N=103)** | **Total (N=206**) |
| Site | | | |
| N* | 103 | 103 | 206 |
| Korle Bu Teaching Hospital (KBTH) | 55 (53%) | 55 (53.4%) | 110 (53.4%) |
| Weija Gbawe Municipal Hospital (WGMH) | 48 (47%) | 48 (46.6%) | 96 (46.6%) |
| Sex | | | |
| N-Miss^†^ | 0 | 1 | 1 |
| N | 103 | 102 | 205 |
| Male | 32 (31%) | 32 (31.4%) | 64 (31.2%) |
| Female | 71 (69%) | 70 (68.6%) | 141 (68.8%) |
| Age (years) | | | |
| N-Miss | 0 | 1 | 1 |
| N | 103 | 102 | 205 |
| Median (Q1^††^, Q3^¶)^ | 57 (50, 64) | 59.00 (49.00, 64.00) | 58.00 (49.00, 64.00) |
| Age Group | | | |
| N-Miss | 0 | 1 | 1 |
| N | 103 | 102 | 205 |
| < 40 years | 8 (8%) | 9 (8.8%) | 17 (8.3%) |
| >= 40 years | 95 (92%) | 93 (91.2%) | 188 (91.7%) |
| Education | | | |
| N-Miss | 1 | 0 | 1 |
| N | 102 | 103 | 205 |
| None | 11 (11%) | 10 (10%) | 21 (10%) |
| Primary | 17 (17%) | 16 (16%) | 33 (16%) |
| Middle or Junior High School | 40 (39%) | 37 (36%) | 77 (38%) |
| Secondary or Senior High School | 22 (22%) | 16 (16%) | 38 (19%) |
| Vocational school | 1 (1%) | 9 (9%) | 10 (5%) |
| Tertiary or higher | 11 (11%) | 15 (15%) | 26 (13%) |
| Occupation | | | |
| N | 103 | 103 | 206 |
| Professionals with degrees | 5 (5%) | 8 (8%) | 13 (6%) |
| Professionals without degrees | 3 (3%) | 10 (10%) | 13 (6%) |
| small scale entrepreneurs | 19 (19%) | 19 (18%) | 38 (18%) |
| small scale farmers | 13 (13%) | 13 (13%) | 26 (13%) |
| Labourers and petty traders | 19 (19%) | 18 (18%) | 37 (18%) |
| educated youth, unemployed | 44 (43%) | 35 (34%) | 79 (38%) |
| Total Income(Dollars) | | | |
| N-Miss | 10 | 8 | 18 |
| N | 93 | 95 | 188 |

^*^Q1 is the lower quartile. ^†^ Q2 is the upper quartile. ^‡^N is number of observations.

^#^% is percentage of observation. ^¶^ N-Miss is number of missing observations **^**^**HbA1c is glycated Haemoglobin.

| **Supplementary Table 1 (b)** continued. Baseline characteristics of participants, by intervention group, median(Q1^*^,Q3^†^) for continuous or N^‡^ (%)^#^ for categorical variables. | | | |
| --- | --- | --- | --- |
|  | **Control (N=103)** | **Intervention (N=103)** | **Total (N=206**) |
| Median (Q1, Q3) | 40 (20, 90) | 50 (23, 100) | 50 (20, 100) |
| Duration of diabetes | | | |
| N | 103 | 103 | 206 |
| Median (Q1, Q3) | 5 (3, 10) | 5 (2, 10) | 5 (3, 10) |
| Blood glucose (mmol/l) | | | |
| N | 103 | 103 | 206 |
| Median (Q1, Q3) | 7.8 (6.1, 9.9) | 8.0 (6.3, 10.4) | 7.9 (6.2, 10.1) |
| HbA1c (%) | | | |
| N | 103 | 103 | 206 |
| Median (Q1, Q3) | 7.6 (6.3, 9.6) | 8.2 (6.5, 10.6) | 7.9 (6.4, 10.1) |
| HbA1c (mmol/mol) | | | |
| N | 103 | 103 | 206 |
| Median (Q1, Q3) | 58 (44, 82) | 67 (47, 92) | 64 (45, 88) |
| Plasma glucose (mmol/l) | | | |
| N-Miss | 0 | 2 | 2 |
| N | 103 | 101 | 204 |
| Median (Q1, Q3) | 7.8 (5.3, 12.3) | 7.6 (5.5, 11.8) | 7.7 (5.4, 12.0) |
| Estimated average glucose (mmol/l) | | | |
| N-Miss | 2 | 5 | 7 |
| N | 101 | 98 | 199 |
| Median (Q1, Q3) | 9.3 (7.4, 12.7) | 10.5 (7.6, 14.4) | 10.0 (7.4, 13.6) |
| Weight (kg) |  |  |  |
| N | 103 | 103 | 206 |
| Median (Q1, Q3) | 78 (68, 86) | 77 (66, 85) | 77 (66, 85) |
| Height (cm) | | | |
| N-Miss | 4 | 2 | 6 |
| N | 99 | 101 | 200 |
| Median (Q1, Q3) | 1.66 (1.59, 1.72) | 1.62 (1.56, 1.71) | 1.64 (1.57, 1.71) |
| Body mass index (kg/m^2^) | | | |
| N-Miss | 4 | 2 | 6 |
| N | 99 | 101 | 200 |
| Median (Q1, Q3) | 28 (24, 32) | 28 (25, 33) | 28 (25, 33) |
| Systolic blood pressure (mmHg) | | | |
| N | 103 | 103 | 206 |
| Median (Q1, Q3) | 127(116, 141) | 128 (113, 138) | 127 (115, 139) |
| Diastolic blood pressure (mmHg) |  |  |  |
| N | 103 | 103 | 206 |
| Median (Q1, Q3) | 79 (74, 87) | 80 (72, 88) | 80 (73, 88) |
| Problem Areas In Diabetes (PAID-5) score | | | |
| N | 103 | 103 | 206 |
| Median (Q1, Q3) | 5 (2, 8) | 4 (1, 7) | 4 (1, 8) |
| Summary of Diabetes Self-care Activities (SDSCA), (days/ week) | | | |
| Healthy eating |  |  | 0.345 |
| N | 103 | 103 | 206 |
| Median (Q1, Q3) | 5(2, 8) | 6.00 (3.00, 8.00) | 6.00 (3.00, 8.00) |
| General diet | | | |
| N | 103 | 103 | 206 |
| Median (Q1, Q3) | 6.00 (1.50, 7.00) | 6.00 (3.00, 8.00) | 6.00 (3.00, 8.00) |
| Fruit and vegetable consumption | | | |
| N-Miss | 2 | 2 | 4 |

^*^Q1 is the lower quartile. ^†^ Q2 is the upper quartile. ^‡^N is number of observations.

^#^% is percentage of observation. ^¶^ N-Miss is number of missing observations **^**^**HbA1c is glycated Haemoglobin

| **Supplementary Table 1 (c)** continued. Baseline characteristics of participants, by intervention group, median(Q1^*^,Q3^†^) for continuous or N^‡^ (%)^#^ for categorical variables. | | | |
| --- | --- | --- | --- |
|  | **Control (N=103)** | **Intervention (N=103)** | **Total (N=206**) |
| N | 101 | 101 | 202 |
| Median (Q1, Q3) | 4.00 (2.00, 6.00) | 4.00 (2.00, 6.00) | 4.00 (2.00, 6.00) |
| High fat food consumption | | | |
| N-Miss | 1 | 2 | 3 |
| N | 102 | 101 | 203 |
| Median (Q1, Q3) | 3.00 (2.00, 5.00) | 2.00 (1.00, 4.00) | 3.00 (1.00, 4.50) |
| Exercise |  |  |  |
| N-Miss | 2 | 4 | 6 |
| N | 101 | 99 | 200 |
| Median (Q1, Q3) | 3.00 (1.00, 7.00) | 4.00 (1.00, 6.00) | 3.00 (1.00, 7.00) |
| Engage in specific exercise |  |  |  |
| N-Miss | 0 | 2 | 2 |
| N | 103 | 101 | 204 |
| Median (Q1, Q3) | 2.00 (1.00, 6.00) | 1.00 (1.00, 4.00) | 1.00 (1.00, 5.00) |
| Test blood sugar |  |  |  |
| N-Miss | 1 | 1 | 2 |
| N | 102 | 102 | 204 |
| Median (Q1, Q3) | 1.00 (1.00, 2.00) | 2.00 (1.00, 2.00) | 2.00 (1.00, 2.00) |
| Test blood sugar as recommended | | | |
| N-Miss | 0 | 1 | 1 |
| N | 103 | 102 | 205 |
| Median (Q1, Q3) | 1.00 (1.00, 1.00) | 1.00 (1.00, 2.00) | 1.00 (1.00, 2.00) |
| Foot care |  |  |  |
| N | 103 | 103 | 206 |
| Median (Q1, Q3) | 1.00 (1.00, 4.00) | 2.00 (1.00, 8.00) | 1.00 (1.00, 7.00) |
| Inspect inside shoes |  |  |  |
| N-Miss | 0 | 1 | 1 |
| N | 103 | 102 | 205 |
| Median (Q1, Q3) | 1.00 (1.00, 1.00) | 1.00 (1.00, 1.00) | 1.00 (1.00, 1.00) |
| Smoked a cigarette |  |  |  |
| N-Miss | 18 | 21 | 39 |
| N | 85 | 82 | 167 |
| Median (Q1, Q3) | 2.00 (2.00, 2.00) | 2.00 (2.00, 2.00) | 2.00 (2.00, 2.00) |
| Quality of life domain scores, median (Q1,Q3) | | | |
| Physical health |  |  | 0.056 |
| N-Miss | 1 | 1 | 2 |
| N | 102 | 102 | 204 |
| Median (Q1, Q3) | 92 (81, 99) | 92 (88, 104) | 92 (84, 100) |
| Psychological |  |  | 0.540 |
| N-Miss | 1 | 2 | 3 |
| N | 102 | 101 | 203 |
| Median (Q1, Q3) | 80 (76, 88) | 80 (76, 88) | 80 (76, 88) |
| Social relationship |  |  |  |
| N | 103 | 103 | 206 |
| Median (Q1, Q3) | 44 (36, 48) | 44 (40, 48) | 44 (36, 48) |
| Environment |  |  |  |
| N-Miss | 1 | 3 | 4 |
| N | 102 | 100 | 202 |
| Median (Q1, Q3) | 112 (100, 124) | 116 (104, 125) | 112 (104, 124) |

| **Supplementary Table 2.** Change in HbA1c ^¶^ (primary outcome) from baseline to follow-up, by intervention group, mean (CI).^**^ | | | | | |
| --- | --- | --- | --- | --- | --- |
| **Arm** | **Baseline (N=206)** | | **Endline (N=206)** | **Difference (N=206)** | **p value** |
| Control | HbA1c-mmol/mol |  |  | 0.172 | |
|  | N-Miss^+^ | 0 | 23 | 23 | |
|  | N^*^ | 103 | 80 | 80 | |
|  | Mean (CI) | 66 (61 to 71) | 60 (55 to 65) | -3 (-6 to 1) | |
|  | HbA1c-% |  |  | 0.068 | |
|  | N-Miss | 0 | 23 | 23 | |
|  | N | 103 | 80 | 80 | |
|  | Mean (CI) | 8.2 (7.7 to 8.7) | 7.7 (7.2 to 8) | -0.3 (-0.6 to 0.0) | |
|  |  |  |  |  | |
| Intervention | HbA1c-mmol/mol |  |  | < 0.001 | |
|  | N-Miss | 0 | 24 | 24 | |
|  | N | 103 | 79 | 79 | |
|  | Mean (CI) | 70 (64 to 75) | 60 (55 to 65) | -9 (-13 to -5) | |
|  | HbA1c-% |  |  | < 0.001 | |
|  | N-Miss | 0 | 24 | 24 | |
|  | N | 103 | 79 | 79 | |
|  | Mean (CI) | 8.5 (8.0 to 9.0) | 7.7 (7.2 to 8.1) | -0.9 (-1.2 to -0.5) | |

^¶^HbA1c is glycated Haemoglobin. ^**^Data are presented as estimates from paired t-tests. ^*^N is number of observations.

^†^N-Miss is number of missing observations.

| **Supplementary Table 3 (a).** Effect of the intervention (structured DSME^#^) on the secondary outcome (average change in SDSCA^¶^) after 3-months follow-up, mean (CI). | | | | | |
| --- | --- | --- | --- | --- | --- |
| **Arm** | **Baseline (N=206)** | | **Endline (N=206)** | **Difference (N=206)** | **p value** |
| Control | Healthy eating |  |  | 0.002 | |
|  | N-Miss^†^ | 0 | 15 | 15 | |
|  | N^*^ | 103 | 88 | 88 | |
|  | Mean (CI) | 5 (4 to 5) | 6 (5 to 6) | 1 (0 to 2) | |
|  | Followed eating plan |  |  | 0.017 | |
|  | N-Miss | 0 | 15 | 15 | |
|  | N | 103 | 88 | 88 | |
|  | Mean (CI) | 5 (4 to 5) | 5 (5 to 6) | 1 (0 to 1) | |
|  | Eat Fruits and vegetable |  |  |  | 0.261 |
|  | N-Miss | 2 | 15 | 17 | |
|  | N | 101 | 88 | 86 | |
|  | Mean (CI) | 4 (3.6 to 4.5) | 4 (4 to 5) | 0 (0 to 1) | |
|  | Eat High fat food |  |  | < 0.001 | |
|  | N-Miss | 1 | 15 | 16 | |
|  | N | 102 | 88 | 87 | |
|  | Mean (CI) | 3 (3 to 4) | 2 (1.8 to 2.5) | -1 (-2 to -1) | |
|  | Exercise past |  |  | 0.003 | |
|  | N-Miss | 2 | 17 | 19 | |
|  | N | 101 | 86 | 84 | |
|  | Mean (CI) | 4 (3 to 4) | 5 (5 to 6) | 1 (0 to 2) | |
| Intervention | Healthy eating |  |  |  | 0.122 |
|  | N-Miss | 0 | 14 | 14 | |
|  | N | 103 | 89 | 89 | |
|  | Mean (CI) | 5 (5 to 6) | 6 (5 to 6) | 1 (0 to 1) | |
|  | Followed eating plan |  |  | 0.162 | |
|  | N-Miss | 0 | 14 | 14 | |
|  | N | 103 | 89 | 89 | |
|  | Mean (CI) | 5 (5 to 6) | 6 (5 to 6) | 0 (0 to 1) | |
|  | Eat Fruits and vegetable |  |  | 0.054 | |
|  | N-Miss | 2 | 14 | 16 | |
|  | N | 101 | 89 | 87 | |
|  | Mean (CI) | 4 (4 to 5) | 5 (4 to 5) | 1 (0 to 1) | |
|  | Eat High fat food |  |  | 0.019 | |
|  | N-Miss | 2 | 14 | 16 | |
|  | N | 101 | 89 | 87 | |
|  | Mean (CI) | 3 (2.6 to 3.4) | 2 (2 to 3) | -1 (-1 to 0) | |
|  | Exercise past |  |  | < 0.001 | |
|  | N-Miss | 4 | 16 | 20 | |
|  | N | 99 | 87 | 83 | |
|  | Mean (CI) | 4 (3 to 4) | 6 (5 to 6) | 2 (1 to 2) | |

^#^DSME is structured diabetes self-management education. ^¶^SDSCA is summary of diabetes self-care assessment.

^**^Data are presented as estimates from paired t-tests. ^*^N is number of observations. ^†^N-Miss is number of missing observations.

| **Supplementary Table 3 (b) continued.** Effect of the intervention (structured DSME^#^) on the secondary outcome (average change in SDSCA^¶^) after 3-months follow-up, mean (CI). | | | | | |
| --- | --- | --- | --- | --- | --- |
| **Arm** | **Baseline (N=206)** | | **Endline (N=206)** | **Difference (N=206)** | **p value** |
| Control | Specific exercise |  |  | 0.351 | |
|  | N-Miss | 0 | 16 | 16 | |
|  | N | 103 | 87 | 87 | |
|  | Mean (CI) | 3 (3 to 4) | 3 (2 to 4) | 0 (-1 to 0) | |
|  | test blood sugar |  |  | 0.829 | |
|  | N-Miss | 1 | 16 | 17 | |
|  | N | 102 | 87 | 86 | |
|  | Mean (CI) | 2 (1.7 to 2.3) | 2 (1.6 to 2.3) | 0 (-0.4 to 0.5) | |
|  | test blood sugar as recommended |  |  | 0.005 | |
|  | N-Miss | 0 | 16 | 16 | |
|  | N | 103 | 87 | 87 | |
|  | Mean (CI) | 2 (1 to 2) | 2 (1.6 to 2.4) | 0.6 (0 to 1) | |
|  | check feet |  |  | 0.003 | |
|  | N-Miss | 0 | 15 | 15 | |
|  | N | 103 | 88 | 88 | |
|  | Mean (CI) | 3 (2 to 3) | 4 (3 to 5) | 1 (0 to 2) | |
|  | Inspect inside shoes? |  |  |  | 0.003 |
|  | N-Miss | 0 | 16 | 16 | |
|  | N | 103 | 87 | 87 | |
|  | Mean (CI) | 2 (1 to 2) | 3 (2 to 3) | 1 (0 to 2) | |
|  | smoked a cigarette |  |  | 0.321 | |
| N-Miss | | 18 | 27 | 39 | |
| N | | 85 | 76 | 64 | |
| Mean (CI) | | 2 (1.99 to 2.01) | 2.00 (2.00, 2.00) | 0.02 (-0.02, 0.05) | |
| Intervention Specific exercise | |  |  | < 0.001 | |
| N-Miss | | 2 | 14 | 16 | |
| N | | 101 | 89 | 87 | |
| Mean (CI) | | 3 (2 to 3) | 4 (4 to 5) | 2 (1 to 2) | |
|  | test blood sugar |  |  | 0.599 | |
|  | N-Miss | 1 | 14 | 15 | |
|  | N | 102 | 89 | 88 | |
|  | Mean (CI) | 2 (2 to 3) | 2 (2 to 3) | 0.1 (-0.3 to 0.5) | |
|  | test blood sugar as recommended |  |  | 0.096 | |
|  | N-Miss | 1 | 14 | 15 | |
|  | N | 102 | 89 | 88 | |
|  | Mean (CI) | 2 (1 to 2) | 2 (1.6 to 2.3) | 0 (0 to 1) | |
|  | check feet |  |  |  | < 0.001 |
|  | N-Miss | 0 | 14 | 14 | |
|  | N | 103 | 89 | 89 | |
|  | Mean (CI) | 4 (3 to 4) | 5 (5 to 6) | 2 (1 to 3) | |
|  | Inspect inside shoes? |  |  | < 0.001 | |
|  | N-Miss | 1 | 14 | 15 | |
|  | N | 102 | 89 | 88 | |
|  | Mean (CI) | 2 (1.5 to 2.3) | 3 (3 to 4) | 2 (1 to 2) | |
|  | smoked a cigarette |  |  | 0.321 | |
|  | N-Miss | 21 | 19 | 36 | |
|  | N | 82 | 84 | 67 | |
|  | Mean (CI) | 2 (2.00, 2.00) | 2 (1.94, 2.01) | 0 (-0.04, 0.01) | |

| **Supplementary Table 3 (c) continued.** Effect of the intervention (structured DSME^#^) on the secondary outcome (clinical variables) after 3-months follow-up, mean (CI).^**^ | | | | | |
| --- | --- | --- | --- | --- | --- |
| **Arm** | **Baseline (N**^*^**=206)** | | **Endline (N=206)** | **Difference (N=206)** | **P-value** |
| Control | Weight (KG) |  |  | 0.144 | |
|  | N-Miss^†^ | 0 | 24 | 24 | |
|  | N^*^ | 103 | 79 | 79 | |
|  | Mean (CI) | 79 (75 to 83) | 79 (75 to 83) | 1 (0 to 2) | |
|  | Waist circumference (cm) |  |  | 0.015 | |
|  | N-Miss | 0 | 24 | 24 | |
|  | N | 103 | 79 | 79 | |
|  | Mean (CI) | 95 (91 to 99) | 100 (97 to 103) | 5 (1 to 8) | |
|  | Average SBP |  |  | 0.143 | |
|  | N-Miss | 0 | 24 | 24 | |
|  | N | 103 | 79 | 79 | |
|  | Mean (CI) | 129 (125 to 132) | 131(127 to 136) | 2.6 (-1, 6) | |
|  | Average DBP |  |  | 0.262 | |
|  | N-Miss | 0 | 24 | 24 | |
|  | N | 103 | 79 | 79 | |
|  | Mean (CI) | 81 (79 to 84) | 79 (77 to 82) | -1 (-4 to 1) | |
|  | PAID |  |  | 0.672 | |
| N | | 103 | 103 | 103 | |
| Mean (CI) | | 6 (5 to 6) | 6 (5 to 7) | 0 (-1 to 1) | |
| Intervention Weight (KG) | |  |  | 0.777 | |
| N-Miss | | 0 | 25 | 25 | |
| N | | 103 | 78 | 78 | |
| Mean (CI) | | 77 (75 to 80) | 77 (74 to 81) | 0 (-1 to 2) | |
|  | Waist circumference (cm) |  |  | 0.688 | |
|  | N-Miss | 0 | 27 | 27 | |
|  | N | 103 | 76 | 76 | |
|  | Mean (CI) | 97 (94 to 100) | 97 (94 to 100) | 0 (-2 to 3) | |
|  | Average SBP |  |  | 0.249 | |
|  | N-Miss | 0 | 25 | 25 | |
|  | N | 103 | 78 | 78 | |
|  | Mean (CI) | 130 (126 to 134) | 132 (128 to 136) | 2 (-2 to 6) | |
|  | Average DBP |  |  |  | 0.902 |
| N-Miss | | 0 | 25 | 25 | |
| N | | 103 | 78 | 78 | |
| Mean (CI) | | 81 (79 to 84) | 81 (78 to 83) | 0 (-3 to 2) | |
|  | PAID |  |  |  | 0.013 |
| N | | 103 | 103 | 103 | |
| Mean (CI) | | 5 (4 to 5) | 6 (5 to 7) | 1 (0 to 2) | |

^#^DSME is structured diabetes self-management education. ^**^Data are presented as estimates from paired t-tests.

N is number of observations. ^†^N-Miss is number of missing observations

| **Supplementary Table 3 (d) continued.** Effect of the intervention (structured DSME^#^) on the secondary outcome (Quality of life^¶^) after 3-months follow-up, mean (CI).^**^ | | | | | |
| --- | --- | --- | --- | --- | --- |
| Arm | Baseline (N=206) | | Endline (N=206) | Difference (N=206) | p value |
| Control | Physical health |  |  | 0.123 | |
|  | N-Miss | 1 | 15 | 16 | |
|  | N | 102 | 88 | 87 | |
|  | Mean (CI) | 90 (87 to 93) | 93 (90 to 96) | 3 (-1 to 6) | |
|  | Psychological |  |  | 0.937 | |
|  | N-Miss | 1 | 15 | 16 | |
|  | N | 102 | 88 | 87 | |
|  | Mean (CI) | 80 (79 to 82) | 81 (79 to 83) | 0 (-2 to 2) | |
|  | Social relationship |  |  | 0.100 | |
|  | N-Miss | 0 | 21 | 21 | |
|  | N | 103 | 82 | 82 | |
|  | Mean (CI) | 43 (42 to 45) | 43 (41 to 44) | -2 (-4 to 0.3) | |
|  | Environment |  |  | 0.558 | |
|  | N-Miss | 1 | 16 | 17 | |
|  | N | 102 | 87 | 86 | |
|  | Mean (CI) | 112 (109 to 115) | 112 (108 to 115) | -1 (-6 to 3) | |
| Intervention | Physical health |  |  |  | 0.753 |
|  | N-Miss | 1 | 14 | 15 | |
|  | N | 102 | 89 | 88 | |
|  | Mean (CI) | 94 (91 to 96) | 95 (93 to 97) | 1 (-3 to 4) | |
|  | Psychological |  |  | 0.282 | |
|  | N-Miss | 2 | 14 | 16 | |
|  | N | 101 | 89 | 87 | |
|  | Mean (CI) | 81 (80 to 83) | 80 (78 to 83) | -1 (-4 to 1) | |
|  | Social relationship |  |  | 0.884 | |
|  | N-Miss | 0 | 20 | 20 | |
|  | N | 103 | 83 | 83 | |
|  | Mean (CI) | 43 (41 to 44) | 43 (41 to 45) | 0 (-2 to 2) | |
|  | Environment |  |  | 0.798 | |
|  | N-Miss | 3 | 15 | 18 | |
|  | N | 100 | 88 | 85 | |
|  | Mean (CI) | 113 (110 to 117) | 113 (110 to 116) | -1 (-5 to 3) | |

^#^DSME is structured diabetes self-management education. ^¶^Quality of life was assessed with the World Health Organisation BREF tool.

^**^Data are presented as estimates from paired t-tests. ^*^N is number of observations. ^†^N-Miss is number of missing observations

| **Supplementary Table 4.** Effect of the intervention (structured DSME^*^) on the average change in PAID-5^†^ after 3-months follow-up. ^‡^ | | | |
| --- | --- | --- | --- |
| **Fixed Effects** | **PAID 5** | | |
| **Parameters** | **Coefficients** | **95% CI** | **P-value** |
| Intercept | 7 | 4.5 to 10.3 | < .001 |
| Site (WGMH) ^¶^ | 1 | -0.1 to 2.1 | 0.085 |
| Diabetes duration (years)* | 0 | -0.1 to 0.1 | 0.777 |
| Age* | 0 | -0.1 to 0.0 | 0.056 |
| Arm (Intervention) | 0 | -1.6 to 0.6 | 0.361 |
| Follow-up interval**^#^** | 0 | 0 .0to 0.5 | 0.054 |
| **Random effects** | | | |
| **Parameter** | **SD** | | |
| Intercept | 3 | | |
| Residual | 4 | | |
| Intraclass correlation | 0.3 | | |

^*^DSME is diabetes self-management education intervention. The intervention tested was an adapted version of an evidence based structured DSME: Diabetes Self-Management Education for New and ON-going Diabetes (DESMOND).[6, 14] The comparator was usual care.

^†^ PAID-5 is Problem Areas in Diabetes Score. It was used to assess diabetes related distress.

^‡^Data are presented as coefficient estimates from linear mixed models.

^¶^WGMH is Weija Gbawe Municipal Hospital site. The comparator was Korle Bu Teaching Hospital Polyclinic.

**^#^** Participants were followed for at least three months.

| **Supplementary Table 4 (b).** Effect of the intervention (structured DSME^*^) on the average change in blood pressure after 3-months follow-up. ^‡^ | | | | | | |
| --- | --- | --- | --- | --- | --- | --- |
| **Fixed Effects** | **SBP**^†^ | | | **DBP**^††^ | | |
| **Parameters** | **Coefficients** | **95% CI** | **P-value** | **Coefficients** | **95% CI** | **P-value** |
| Intercept | 97 | 85 to 108 | < .001 | 82 | 75 to 90 | < .001 |
| Site (WGMH) ^¶^ | -2.3 | 7 to 2 | 0.085 | -2.4 | -5.3 to 0.5 | 0.108 |
| Diabetes duration (years)* | 0.2 | 0 to 1 | 0.777 | 0.0 | -0.3 to 0.2 | 0.732 |
| Age* | 0.6 | 0 to 1 | 0.056 | 0.0 | -0.1 to 0.1 | 0.904 |
| Arm (Intervention) | 1.1 | -3 to 6 | 0.361 | 0.3 | -2.6 to 3.2 | 0.830 |
| Follow-up interval**^#^** | 0.8 | -0 to 2 | 0.054 | 0.3 | -0.8 to 0.2 | 0.256 |
|  | **Random Effects** | | | | | |
| **Parameter** | **SD** | | | **SD** | | |
| Intercept | 13 | | | 8.5 | | |
| Residual | 12 | | | 8.8 | | |
| Intraclass correlation | 0.6 | | | 0.5 | | |

^*^DSME is diabetes self-management education intervention. The intervention tested was an adapted version of an evidence based structured DSME: Diabetes Self-Management Education for New and ON-going Diabetes (DESMOND).[6, 14] The comparator was usual care.

^†^ SBP is mean systolic blood pressure. ^††^ DBP is mean diastolic blood pressure.

^‡^Data are presented as coefficient estimates from linear mixed models.

^¶^WGMH is Weija Gbawe Municipal Hospital site. The comparator was Korle Bu Teaching Hospital Polyclinic.

**^#^** Participants were followed for at least three months.

| **Supplementary Table 4 (c).** Effect of the intervention (structured DSME^*^) on the average change in waist circumference and weight after 3-months follow-up. ^‡^ | | | | | | |
| --- | --- | --- | --- | --- | --- | --- |
| **Fixed Effects** | **Waist circumference** | | | **Weight** | | |
| **Parameters** | **Coefficients** | **95% CI** | **P-value** | **Coefficients** | **95% CI** | **P-value** |
| Intercept | 81 | 70 to 92 | < .001 | 78 | 66 to 90 | < .001 |
| Site (WGMH) ^¶^ | -3.71 | -7.8 to 0.4 | 0.078 | 0.2 | -4.6 to 4.9 | 0.950 |
| Diabetes duration (years)* | 0.0 | -0.3 to 0.4 | 0.912 | 0.1 | -0.3 to 0.4 | 0.771 |
| Age* | 0.29 | 0.1 to 0.5 | 0.002 | 0.0 | -0.2 to 0.2 | 0.933 |
| Arm (Intervention) | 0.10 | -4.0 to 4.2 | 0.962 | -2.2 | -7.0 to 2.5 | 0.351 |
| Follow-up interval**^#^** | 0.85 | 0 .1 to 1.6 | 0.023 | 0.2 | -0.1 to 0.5 | 0.246 |
|  | **Random Effects** | | | | | |
| **Parameter** | **SD** | | | **SD** | | |
| Intercept | 13 | | | 17 | | |
| Residual | 9.9 | | | 4.2 | | |
| Intraclass correlation | 0.6 | | | 0.9 | | |

^*^DSME is diabetes self-management education intervention. The intervention tested was an adapted version of an evidence based structured DSME: Diabetes Self-Management Education for New and ON-going Diabetes (DESMOND).[6, 14] The comparator was usual care.

^†^ HbA1c is glycated Haemoglobin.

^‡^Data are presented as coefficient estimates from linear mixed models.

^¶^WGMH is Weija Gbawe Municipal Hospital site. The comparator was Korle Bu Teaching Hospital Polyclinic.

**^#^** Participants were followed for at least three months.

| **Supplementary Table 4 (d).** Effect of the intervention (structured DSME^*^) on the average change in quality of life after 3-months follow-up. ^‡^ | | | | | | |
| --- | --- | --- | --- | --- | --- | --- |
| **Fixed Effects** | **Physical Health**^†^ | | | **Psychological health**^††^ | | |
| **Parameters** | **Coefficients** | **95% CI** | **P-value** | **Coefficients** | **95% CI** | **P-value** |
| Intercept | 93 | 87 to 100 | < .001 | 80 | 75 to 85 | < .001 |
| Site (WGMH) ^¶^ | 2 | -1 to 5 | 0.175 | -1.1 | -3.0 to 0.8 | 0.253 |
| Diabetes duration (years)* | 0 | -0.4 to 0.0 8 | 0.059 | -0.1 | -0.2 to 0.1 | 0.394 |
| Age* | 0 | -0.2 to 0.01 | 0.462 | 0.0 | -0.1 to 0.1 | 0.469 |
| Arm (Intervention) | 3 | 0. to 5 | 0.035 | 0.3 | -1.7 to 2.2 | 0.796 |
| Follow-up interval**^#^** | 1 | 0 to 1 | 0.081 | -0.2 | -0.8 to 0.4 | 0.487 |
|  | Random Effects | | | | | |
| **Parameter** | SD | | | SD | | |
| Intercept | 4.5 | | | 3.2 | | |
| Residual | 11.4 | | | 8.3 | | |
| Intraclass correlation | 0.1 | | | 0.1 | | |

^*^DSME is diabetes self-management education intervention. The intervention tested was an adapted version of an evidence based structured DSME: Diabetes Self-Management Education for New and ON-going Diabetes (DESMOND).[6, 14] The comparator was usual care.

^†^ Physical health is physical health sub-scale on the World Health Organisation quality of life BREF instrument.

^††^Psychological health is psychological health sub-scale on the World Health Organisation quality of life BREF instrument.

^‡^Data are presented as coefficient estimates from linear mixed models.

^¶^WGMH is Weija Gbawe Municipal Hospital site. The comparator was Korle Bu Teaching Hospital Polyclinic.

**^#^** Participants were followed for at least three months.

| **Supplementary Table 4 (e).** Effect of the intervention (structured DSME^*^) on the average change in quality of life after 3-months follow-up. ^‡^ | | | | | | |
| --- | --- | --- | --- | --- | --- | --- |
| **Fixed Effects** | **Social relationship**^†^ | | | **Environment**^††^ | | |
| **Parameters** | **Coefficients** | **95% CI** | **P-value** | **Coefficients** | **95% CI** | **P-value** |
| Intercept | 45 | 40 to 50 | < .001 | 104 | 95 to 114 | < .001 |
| Site (WGMH) ^¶^ | -0.7 | -2.4 to 1.1 | 0.461 | 1.3 | -2.3 to 4.9 | 0.253 |
| Diabetes duration (years)* | 0.0 | -0.2 to 0.1 | 0.948 | 0.1 | -0.2 to 0.4 | 0.394 |
| Age* | 0.0 | -0.1 to 0.0 | 0.360 | 0.1 | -0.1 to 0.3 | 0.469 |
| Arm (Intervention) | 0.2 | -1.6 to, 1.9 | 0.861 | 2.1 | -1.5 to 5.7 | 0.796 |
| Follow-up interval**^#^** | 0.0 | -0.5 to 0.4 | 0.706 | -0.1 | -1.1 to 0.8 | 0.487 |
|  | Random Effects | | | | | |
| **Parameter** | SD | | | SD | | |
| Intercept | 4.2 | | | 8.4 | | |
| Residual | 6.4 | | | 13 | | |
| Intraclass correlation | 0.3 | | | 0.3 | | |

^*^DSME is diabetes self-management education intervention. The intervention tested was an adapted version of an evidence based structured DSME: Diabetes Self-Management Education for New and ON-going Diabetes (DESMOND).[6, 14] The comparator was usual care.

^†^ Social relationship is the social relationship sub-scale on the World Health Organisation quality of life BREF instrument.

^††^Environment is the environment sub-scale on the World Health Organisation quality of life BREF instrument.

^‡^Data are presented as coefficient estimates from linear mixed models.

^¶^WGMH is Weija Gbawe Municipal Hospital site. The comparator was Korle Bu Teaching Hospital Polyclinic.

**^#^** Participants were followed for at least three months.
