# Supplementary material for: Change in glycaemic control with structured diabetes self-management education in urban low-resource settings: multicentre randomised trial of effectiveness
Source: BMC Health Serv Res. 2023 Feb 24;23:199. doi: 10.1186/s12913-023-09188-y (PMC9957611; doi:10.1186/s12913-023-09188-y)
Supplement: Supplementary file 2 — Additional file 2. Supplementary figure 2 [file 12913_2023_9188_MOESM2_ESM.docx]

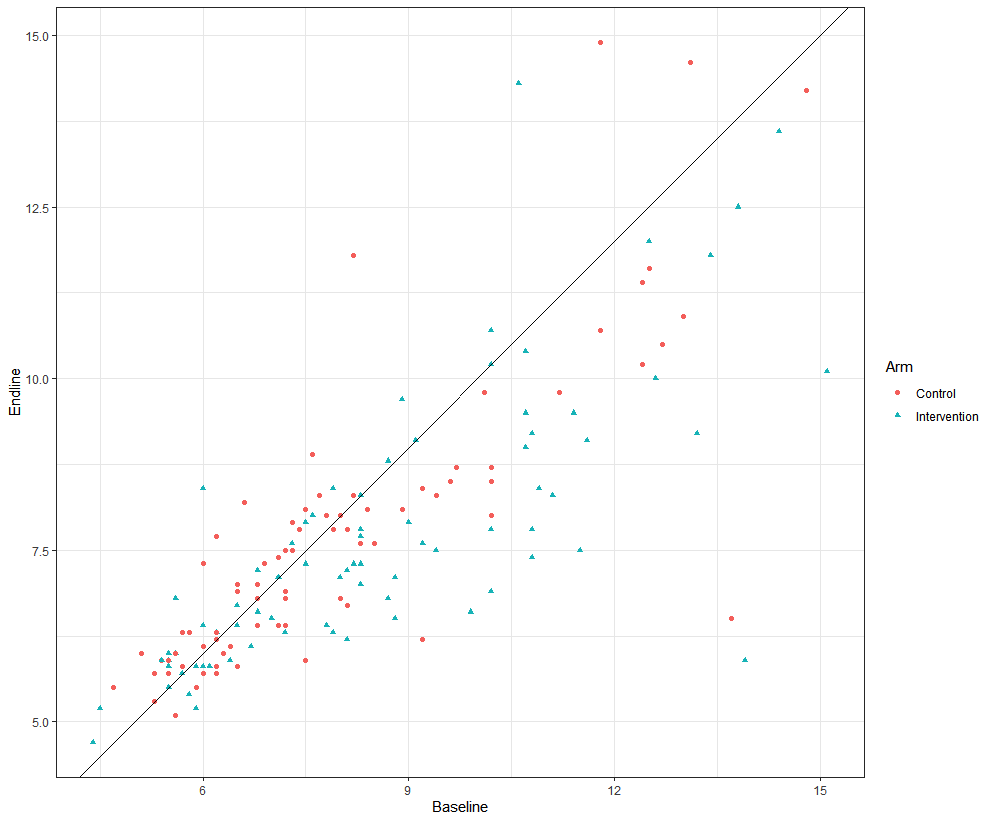


Supplementary Figure 1: A scatter plot of baseline and endline HbA1c for treatment and control with line of equality superimposed.
