# Supplementary figures and images for: Change in glycaemic control with structured diabetes self-management education in urban low-resource settings: multicentre randomised trial of effectiveness
Source: BMC Health Serv Res. 2023 Feb 24;23:199. doi: 10.1186/s12913-023-09188-y (PMC9957611; doi:10.1186/s12913-023-09188-y)

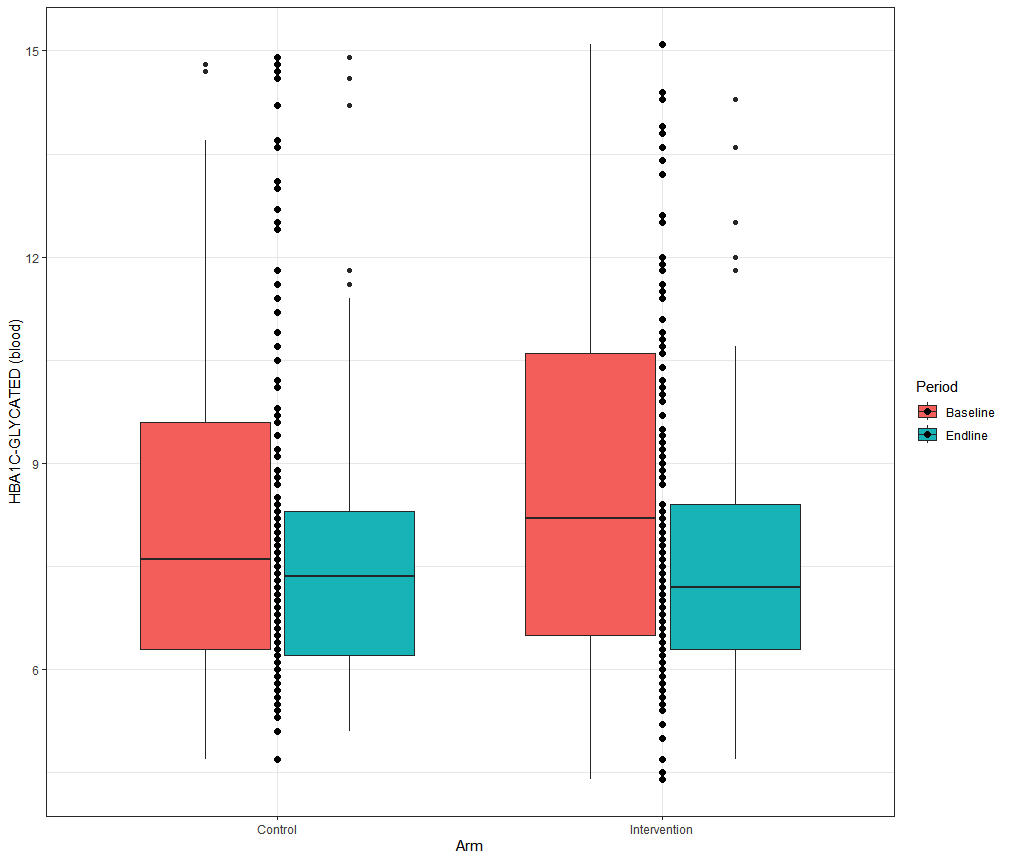


Supplementary Figure 2: A boxplot of baseline and endline HbA1c by treatment and control groups.

Supplement: Supplementary file 3 — Additional file 3. Supplementary tables [file 12913_2023_9188_MOESM3_ESM.docx]
